# Quantitative Ultrasound Assessment of Skeletal Muscle Microvascularity in Relation to Serum 25-Hydroxyvitamin D Concentrations

**DOI:** 10.3390/diagnostics16030369

**Published:** 2026-01-23

**Authors:** Nusret Seher, Lütfiye Parlak, Halil Özer, Esra Öz, Mehmet Sedat Durmaz, Mustafa Koplay

**Affiliations:** 1 Department of Radiology, Medical Faculty, Selcuk University, 42130 Konya, Turkey; 2 Department of Physical Medicine and Rehabilitation, Medical Faculty, Selcuk University, 42130 Konya, Turkey

**Keywords:** ultra micro angiography, ultrasound, vitamin D, skeletal muscle

## Abstract

**Background/Objective:** Vitamin D plays an important role in musculoskeletal health; however, its association with skeletal muscle microvascular perfusion has not been clearly defined using quantitative imaging techniques. To investigate the relationship between serum 25-hydroxyvitamin D [25(OH)D] levels and skeletal muscle microvascularity using an advanced ultrasound-based microvascular imaging method. **Methods:** In this cross-sectional study a total of 141 healthy adults were stratified into two groups according to serum 25(OH)D concentration (<20 ng/mL, *n* = 71; ≥20 ng/mL, *n* = 70). Ultra-Micro-Angiography was used to quantitatively assess the vascular index (VI), while cross-sectional area (CSA) measurements were obtained for the flexor carpi radialis, biceps brachii, and tibialis anterior muscles. Group comparisons and receiver operating characteristic (ROC) analyses were performed to evaluate the discriminative performance of microvascular parameters. **Results:** CSA values did not differ significantly between the groups. In contrast, VI values were significantly higher in participants with higher serum 25(OH)D levels across all examined muscles (*p* < 0.001). Among the evaluated parameters, the biceps brachii VI demonstrated excellent diagnostic performance in distinguishing between groups (AUC = 0.992; optimal cut-off = 1.25). **Conclusions:** Serum 25(OH)D levels are strongly associated with skeletal muscle microvascularity independent of muscle size. These findings demonstrate a strong association between serum 25-hydroxyvitamin D concentration and skeletal muscle microvascularity and highlight the potential of ultra-micro-angiography as a non-invasive imaging approach for detecting microvascular differences related to vitamin D status.

## 1. Introduction

Vitamin D is primarily produced in the skin upon exposure to ultraviolet (UV) radiation, although it can also be obtained through dietary intake. Once synthesized or obtained through diet, vitamin D is first hydroxylated in the liver, producing 25-hydroxyvitamin D [25(OH)D]—the principal form present in circulation—and then further hydroxylated in the kidney to yield the hormonally active 1,25-dihydroxyvitamin D [1]. Additionally, 25(OH)D can be locally activated to calcitriol by 1α-hydroxylase in many extra-renal tissues, including skeletal muscle. According to the most recent published guidelines, serum concentrations of 25(OH)D below 20 ng/mL are defined as Vitamin D deficiency [1,2], whereas serum levels between 20 and 50 ng/mL are commonly regarded as an acceptable range for musculoskeletal health in adults. Vitamin D deficiency has been associated with reduced muscle strength, impaired physical performance, and degenerative muscle changes, particularly in elderly individuals and those with chronic systemic disease [1,2,3,4,5,6,7]. Beyond its musculoskeletal effects, 25(OH)D is also involved in maintaining vascular integrity and cardiovascular homeostasis. Low 25(OH)D levels have been linked to endothelial dysfunction and impaired vascular reactivity, suggesting a potential role in microvascular regulation [8,9,10].

Emerging evidence suggests that vitamin D may also exert direct biological effects on the microvascular system through several molecular pathways. Vitamin D has been shown to modulate endothelial nitric oxide synthase (eNOS) activity, increase nitric oxide (NO) bioavailability, and influence vascular smooth muscle cell function, thereby improving vasodilation and microcirculatory responsiveness [11,12]. Experimental data further suggest that vitamin D may influence angiogenic signaling pathways, supporting a role in microvascular remodeling [13]. Within skeletal muscle, these pathways collectively support the hypothesis that vitamin D may influence microvascular perfusion primarily through modulation of endothelial function and angiogenic signaling rather than through direct effects on muscle fiber structure.

The microvascular network is essential for oxygen delivery, metabolic support, and contractile efficiency. Impaired microvascular perfusion contributes to early fatigability, insulin resistance, and diminished exercise tolerance [14]. Thus, determinants of microvascularity—including vitamin D status—may have substantial implications for muscle health in both healthy and diseased populations.

Ultra Micro Angiography (UMA) is a novel Doppler ultrasound technique designed to enhance the visualization of vascular structures on ultrasonography (US) without the use of contrast agents. Compared with conventional Doppler, UMA employs a wall-filtering algorithm capable of distinguishing slow tissue motion signals from blood flow signals, thereby achieving greater sensitivity to low-velocity blood flow. This enhanced sensitivity allows assessment of microvascular perfusion in settings where conventional Doppler techniques are limited [15,16].

Recent advances in microvascular imaging techniques have expanded the application of UMA in clinical and experimental research. Studies have demonstrated the capability of UMA to detect small-caliber vessels with improved clarity, reduced motion artifacts, and enhanced frame rates compared with conventional Doppler modalities [17,18].

Despite growing interest in vitamin D physiology and its systemic vascular effects, there remains a notable gap in understanding how serum 25(OH)D concentration influences microvascular perfusion in skeletal muscle. To date, no previous study has directly investigated the association between serum 25(OH)D concentration and skeletal muscle microvascular perfusion using advanced ultrasound techniques specifically designed to detect low-velocity microvascular flow.

Assessing skeletal muscle microvascularity in healthy adults may offer clinically relevant insight into early, subclinical vascular changes associated with vitamin D status, before the development of overt musculoskeletal or metabolic impairment. Therefore, in this study, we aimed to quantitatively evaluate skeletal muscle microvascularity and muscle area using ultrasound and Ultra Micro Angiography in healthy individuals stratified according to serum 25(OH)D concentrations.

## 2. Materials and Methods

### 2.1. Study Design and Participants

Following the approval of the university ethics committee (Approval No: 2025/45), this cross-sectional study was conducted between March and June 2025. The study group consisted of participants who presented to the physical therapy and ultrasound outpatient clinics. The research protocol was developed in alignment with the ethical principles set forth in the Declaration of Helsinki. Individuals were excluded from the study if they met any of the following criteria: patients using medications that may cause rhabdomyolysis, those with acute trauma, neuromuscular diseases, individuals engaged in high-intensity physical activity or using drugs for muscle development, chronic disease (e.g., cardiovascular, metabolic, inflammatory, or neuromuscular conditions), pregnancy or lactation, malignancy, or life-threatening conditions such as congenital heart disease. To minimize seasonal variation, all measurements were performed between March and June, when sunlight exposure is relatively stable in central Anatolia. None of the participants were professional athletes or engaged in regular outdoor exercise. The first group consisted of 71 participants with 25(OH)D concentration below 20 ng/mL, while the second group included 70 participants with 25(OH)D concentration ≥20 ng/mL. Serum 25-hydroxyvitamin D concentrations were analyzed using a chemiluminescent immunoassay (Abbott Architect i2000SR, Abbott Diagnostics, Abbott Park, IL, USA). Internal and external quality control procedures were applied according to the manufacturer’s recommendations. Written informed consent was obtained from all participants. At the time of enrollment, demographic information, conventional ultrasound variables, and UMA data for all participants were obtained from the Hospital Information System. A standardized enrollment workflow minimized selection bias. A brief physical examination ensured the absence of recent exertion-induced muscle fatigue. Body mass index (BMI), dominant extremity, and self-reported sun exposure were recorded to ensure sample comparability.

### 2.2. Ultrasound Examination

**Figure 1:**
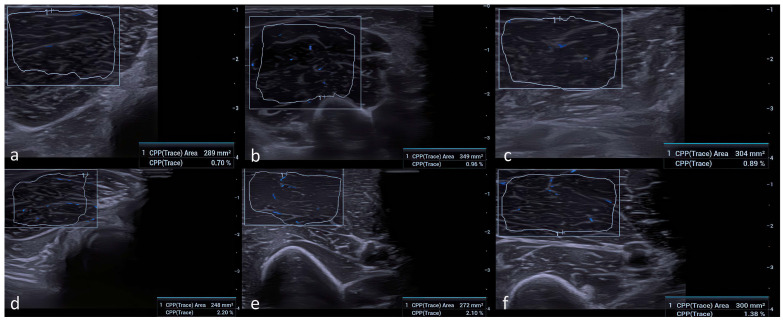
Representative UMA images from participants with different serum 25(OH)D concentrations (ng/mL). For each muscle, the vascular index (VI) represents the ratio of color pixels to the total number of pixels within the ROI. (**a**) FCR muscle: 25(OH)D = 10 ng/mL, VI = 0.7. (**b**) BB muscle: 25(OH)D = 15 ng/mL, VI = 0.96. (**c**) TA muscle: 25(OH)D = 12 ng/mL, VI = 0.89. (**d**) FCR muscle: 25(OH)D = 41 ng/mL, VI = 2.2. (**e**) BB muscle: 25(OH)D = 37 ng/mL, VI = 2.1. (**f**) TA muscle: 25(OH)D = 29 ng/mL, VI = 1.38.

All volunteers included in the study were examined by two independent radiologists (with 9 and 18 years of professional experience, respectively), blinded to the participants’ medical history, surgical information, and clinical variables. Participants were recruited using a consecutive sampling approach from individuals referred for routine ultrasound examinations who met the inclusion criteria. Participants were instructed to avoid strenuous activity for at least 24 h prior to imaging, and examinations were performed with the muscles in a relaxed position at room temperature (22–24 °C). Grayscale ultrasound and Ultra-Micro-Angiography (UMA) examinations were performed using a high-frequency (14 MHz) linear array transducer (Mindray Resona I9, Mindray Medical Systems, Shenzhen, China). Imaging was conducted on the dominant extremities of the participants in the supine normoanatomic position, and participants were instructed to remain still to minimize motion artifacts. Copious coupling gel was applied to reduce subcutaneous tissue-related variability. The transducer was positioned perpendicular to the thickest mid-portion of the flexor carpi radialis (FCR), biceps brachii short head (BB), and tibialis anterior (TA) muscles. Cross-sectional area (CSA) measurements and vascular index (VI) values were obtained at these standardized locations using UMA (Figure 1). The vascular index (VI) was calculated as the ratio of color pixels to the total number of pixels within the selected region of interest (ROI), expressed as a percentage (VI = [color pixels/total pixels] × 100). To avoid artifacts, the ultrasound probe was held steady for 3–5 s, and VI values were recorded once UMA images stabilized. All grayscale ultrasound and UMA parameters were standardized and kept identical for all participants. UMA settings—including pulse repetition frequency (PRF), gain, frame rate, and wall filter thresholds—were held constant throughout the examinations. PRF was set at 600–800 Hz, the wall filter was set to the lowest available level, and gain was optimized to avoid blooming artifacts, ensuring that VI measurements reflected true microvascular signals rather than noise. In practice, PRF was initially set at the lowest level allowing stable signal detection and was minimally adjusted when necessary to avoid motion artifacts; all final PRF values remained within the predefined 600–800 Hz range across participants. All UMA sensitivity settings, including gain, pulse repetition frequency, and wall filter parameters, were fixed and identical across participants and muscle groups, minimizing the likelihood that technical variability or operator-dependent factors contributed to systematic inflation of vascular index measurements. To ensure measurement reproducibility, all CSA and VI measurements were performed twice and averaged. Inter- and intra-observer reliability was assessed in a randomly selected subset of 20 participants, yielding excellent agreement (ICC: 0.91–0.96 for CSA and 0.88–0.94 for VI).

### 2.3. Statistical Analysis

Data analyses were carried out using IBM SPSS Statistics software, version 21.0 (IBM Corp., Armonk, NY, USA). Normality of continuous variables was assessed using the Kolmogorov–Smirnov test and visual inspection of distribution histograms. Non-normally distributed numerical data were summarized as median (min–max), and categorical data were expressed as counts and percentages. Group comparisons for categorical variables (e.g., sex) were conducted using the Chi-square test, while age and vascular index (VI) comparisons were performed using the Mann–Whitney U test. Effect sizes for non-parametric tests were calculated using the rank-biserial correlation to facilitate interpretation of clinical relevance. Diagnostic accuracy was evaluated using receiver operating characteristic (ROC) curve analysis. When the area under the curve (AUC) reached statistical significance, the Youden index was used to determine the optimal cut-off value, which should be interpreted with caution given the exploratory nature of the analysis and the absence of external validation. Sensitivity and specificity were subsequently calculated. A two-tailed *p*-value < 0.05 was considered statistically significant. The relationship between vascular index (VI) and serum 25-hydroxyvitamin D concentration was assessed using Spearman’s rank correlation analysis. No a priori sample size calculation was performed; the sample size was determined by consecutive recruitment of eligible participants during the predefined study period. An exploratory post hoc power analysis based on the observed VI effect size suggested that the sample size (*n* = 141) provided more than 90% statistical power at an alpha level of 0.05. Additionally, multivariable linear regression analyses were performed to examine whether age, sex, body mass index, and muscle cross-sectional area confounded the relationship between vascular index and serum 25(OH)D concentration. To further address the potential confounding effect of age, multivariable linear regression analyses were performed with vascular index as the dependent variable and age, sex, body mass index, and muscle cross-sectional area entered simultaneously as covariates.

## 3. Results

**Figure 2:**
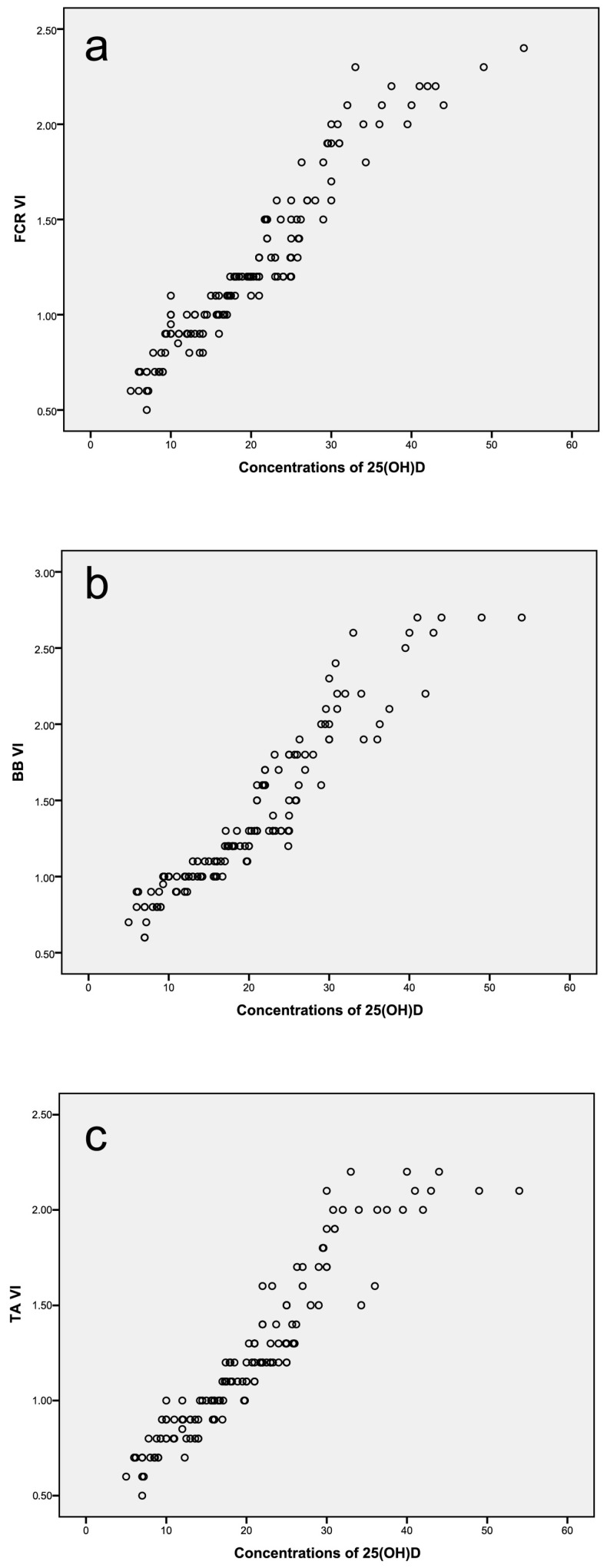
Scatter plots demonstrating the positive linear correlation between serum 25(OH)D concentration (ng/mL) and vascular index (VI) values of the flexor carpi radialis (FCR) (**a**), biceps brachii (BB) (**b**), and tibialis anterior (TA) (**c**) muscles. Spearman correlation coefficients were r = 0.953, r = 0.942, and r = 0.941, respectively (all *p* < 0.001).

**Figure 3:**
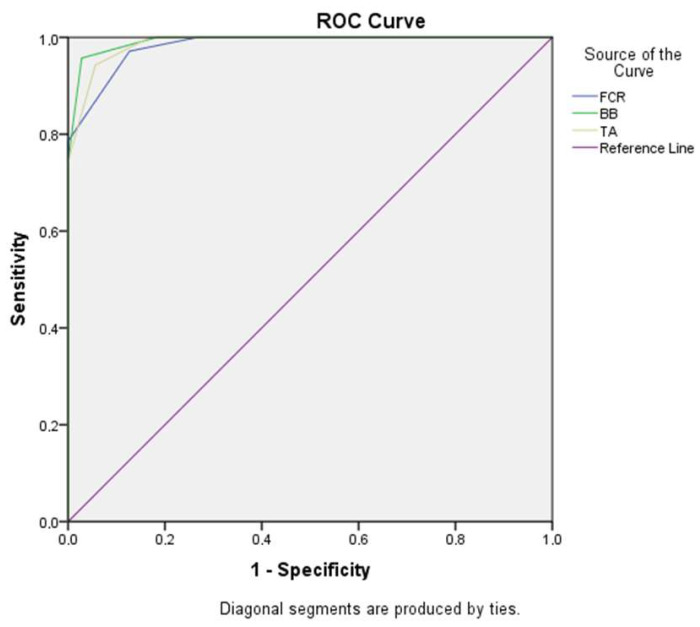
Receiver operating characteristic (ROC) curves showing the discriminative performance of the ultra-micro-angiography–derived vascular index (VI) of the flexor carpi radialis (FCR), biceps brachii (BB), and tibialis anterior (TA) muscles for serum 25-hydroxyvitamin D concentrations < 20 ng/mL and ≥20 ng/mL. The diagonal line indicates no discriminative ability.

Participants were stratified into two groups according to serum 25-hydroxyvitamin D concentration (<20 ng/mL and ≥20 ng/mL). A total of 141 participants with a median age of 28 years (range: 19–47) were included in the study. Table 1 presents the demographic characteristics of the participants. There was no significant difference between the groups in terms of sex distribution (*p* = 0.644); however, age differed significantly between the groups (*p* < 0.001). To evaluate whether this age difference confounded the observed microvascular findings, age-adjusted multivariable regression analyses were performed. These analyses demonstrated that serum 25-hydroxyvitamin D concentration remained independently associated with vascular index across all examined muscles (all *p* < 0.001), and inclusion of age as a covariate did not materially alter the strength or significance of the associations. Detailed regression coefficients are not shown, as adjustment did not materially alter the unadjusted group comparisons. Detailed regression coefficients are not presented, as covariate adjustment did not meaningfully change the direction, magnitude, or statistical significance of the unadjusted group comparisons. The UMA-derived vascular index (VI) values of the flexor carpi radialis, biceps brachii, and tibialis anterior muscles differed significantly between the groups (Table 2, all *p* < 0.001). Figure 2 illustrates the association between serum 25-hydroxyvitamin D concentration and vascular index across all participants. The results of the receiver operating characteristic (ROC) analysis are shown in Figure 3. Among the evaluated parameters, the VI of the biceps brachii muscle demonstrated the highest discriminative performance between groups, with an AUC of 0.992 (*p* < 0.001). A cut-off value of 1.25 yielded a sensitivity of 95.7% and a specificity of 97.2% (Table 3). Significant positive correlations were observed between serum 25-hydroxyvitamin D concentration and vascular index values for all examined muscles (Spearman’s ρ = 0.953 for FCR, 0.942 for BB, and 0.941 for TA; all *p* < 0.001). CSA values showed minimal variation between groups and did not correlate with either VI or serum 25-hydroxyvitamin D concentration, indicating that microvascular differences were independent of muscle size. Adjustment for age did not materially alter the observed associations, and sex-stratified analyses demonstrated similar trends in both males and females.

## 4. Discussion

In this cross-sectional study, we evaluated the relationship between serum 25-hydroxyvitamin D concentration and skeletal muscle microvascular perfusion and demonstrated that muscle vascularity was significantly higher in participants with serum 25(OH)D concentrations ≥ 20 ng/mL. Importantly, no significant differences were observed between the groups in terms of muscle cross-sectional area, suggesting that the observed differences in microvascularity were not driven by structural muscle changes. Although numerous studies have reported the effects of vitamin D on skeletal muscle, demonstrating this relationship using an imaging-based approach provides additional and complementary evidence. The assessment of skeletal muscle microvascularity using imaging techniques—particularly a dynamic method such as Ultra-Micro-Angiography—offers several advantages. UMA is a non-invasive technique capable of visualizing low-velocity microcirculatory flow in real time and can detect subtle changes in muscle vascularity that may not be apparent with conventional imaging modalities. Moreover, UMA is relatively inexpensive, safe, and widely applicable. The ability of UMA to quantitatively assess low-velocity microcirculatory flow provides a novel perspective on vitamin D–related physiological alterations that have traditionally been inferred indirectly through biochemical markers or functional assessments. From a methodological perspective, the relatively narrow range of CSA values observed in our study likely reflects the strict exclusion of athletes, individuals with chronic systemic disease, and participants with recent strenuous physical activity, as well as the relatively young and homogeneous study population. This controlled design may explain the limited inter-individual variability in muscle size and supports the interpretation that microvascular differences occurred independently of muscle morphology.

The UMA technique is a recently developed ultrasound modality designed to assess slow-flow hemodynamics in microvessels. Owing to its high-resolution imaging capability, it provides enhanced sensitivity relative to traditional Doppler sonography. Technically, UMA employs unfocused wave imaging and advanced wall-filtering algorithms, which improve the sensitivity of Doppler ultrasound in detecting microvessels and optimize imaging performance. In addition, flow quantification can be performed using the vascular index (VI), which represents the ratio of points with flow signals to all points in the examined area [15,16]. In the literature, UMA has been reported to be effective in demonstrating microvascularity in renal lesions, infarctions, and renal failure [15,19]. Moreover, a limited number of studies have demonstrated its use in evaluating the liver in biliary atresia, the bowel in inflammatory bowel disease, and the synovium in rheumatoid arthritis, suggesting that UMA may contribute to the assessment of microvascularity [16,20,21]. In the present study, we evaluated microvascularity in selected muscle groups using UMA and found a relationship between 25(OH)D concentration and VI values. The strong interobserver consistency observed in our measurements further demonstrates that UMA is not only sensitive but also highly reproducible in musculoskeletal applications, highlighting its feasibility for broader clinical use.

Vitamin D functions as a steroid hormone essential for mineral metabolism—particularly calcium and phosphorus—and for sustaining normal bone and muscle physiology [1]. Vitamin D is obtained through sunlight exposure, diet, and supplementation [22]. Vitamin D influences muscle tissue via genomic and non-genomic mechanisms, both of which are regulated by receptors located on or within muscle fibers. Some molecular studies have demonstrated that Vitamin D influences muscle cell differentiation, intracellular calcium utilization, and genomic activity [4]. Furthermore, a recent systematic review focusing on healthy adults indicated that higher 25(OH)D concentration may have beneficial effects on increasing muscle strength and reducing injury incidence. Additionally, numerous studies have shown that Vitamin D deficiency is associated with reduced grip strength and impaired muscle performance [4,5,23,24]. Our findings extend these observations by suggesting that the musculoskeletal effects of vitamin D may be mediated, at least in part, through alterations in skeletal muscle microvascular perfusion. Unlike functional test outcomes, vascular index (VI) values represent a quantitative microvascular parameter that may reflect early, subclinical physiological changes. This may explain why significant differences in VI were observed between groups despite the absence of measurable differences in muscle cross-sectional area. Conceptually, microvascular alterations may represent an earlier and more sensitive physiological response to variations in vitamin D status, whereas structural changes in muscle size are likely to occur later and require prolonged or more severe deficiency. All ultrasound measurements were performed using fixed and identical sensitivity settings across participants, reducing the likelihood that the strength of the observed associations was driven by technical artifacts. Given the cross-sectional design of the study, these findings should be interpreted as associations rather than evidence of direct physiological effects or causal mechanisms.

Epidemiological data from U.S. cohorts have demonstrated that lower serum 25(OH)D concentrations are associated with an increased risk of peripheral arterial disease [25,26,27,28]. Vitamin D appears to exert a beneficial effect on arterial blood pressure through the negative regulation of the renin–angiotensin system [27]. Several animal and human studies have reported that Vitamin D supplementation may accelerate vascular repair by increasing the number of angiogenic myeloid cells and other factors, as well as by improving endothelial function [9,10,29,30]. Vitamin D modulates vascular homeostasis by stimulating endothelial nitric oxide synthase (eNOS) expression and activity, which in turn augments nitric oxide generation. This mechanism supports vasodilation and may contribute to the prevention of vascular disorders, including erectile dysfunction [13,27,31,32]. Consistent with its vascular effects, vitamin D deficiency has also been associated with a greater likelihood of erectile dysfunction in clinical studies [33,34]. Taken together, these findings provide a biological context for the observed association between lower serum 25(OH)D concentrations and reduced skeletal muscle microvascular perfusion in the present study. The strong correlations observed across all examined muscle groups further suggest that vitamin D–related effects on skeletal muscle microvascularity may be mediated through systemic endothelial pathways rather than localized muscle-specific mechanisms.

Structural muscle abnormalities, including atrophy and fatty replacement, have been associated with vitamin D deficiency and are readily detectable by cross-sectional imaging techniques. Magnetic resonance imaging findings have confirmed the presence of such degenerative changes in patients with insufficient vitamin D levels [35]. According to the findings of Visser et al., dual-energy X-ray absorptiometry (DEXA)-based assessments revealed that subjects whose serum 25(OH)D concentration was below 25 ng/mL presented with decreased skeletal muscle mass [36]. Quantitative muscle measurements by US have been shown to be a potential alternative for assessing disease severity and progression in various neuromuscular disorders. Additional advantages of US include real-time imaging, low cost, rapid application, and the absence of ionizing radiation [4]. Studies conducted in patients with multiple organ failure and sepsis have demonstrated significant muscle degeneration using US [6,37]. Although many studies have reported reduced muscle performance in Vitamin D deficiency, a limited number of studies including healthy participants without comorbidities—where skeletal muscle thickness was evaluated by US—have shown no significant association between muscle thickness and 25(OH)D concentration [5,7]. Consistent with these findings, our analysis of healthy individuals without comorbidities revealed no statistically significant variation in muscle thickness between subgroups defined by serum 25(OH)D concentration (<20 ng/mL vs. ≥20 ng/mL). However, UMA measurements of muscle vascularity demonstrated significantly higher perfusion in the group with 25(OH)D concentration > 20 ng/mL. This finding aligns with the concept that microvascular alterations often precede measurable structural changes in muscle tissue. These effects may be related to the beneficial influence of vitamin D on endothelial function and its role in promoting vasodilatory mediators such as nitric oxide. Notably, no significant differences in muscle vascularity were observed among participants with serum 25(OH)D concentrations ≥ 50 ng/mL. This observation may represent a hypothesis-generating finding suggesting a potential plateau effect, rather than a definitive threshold, and should be interpreted cautiously given the cross-sectional design of the study. Future studies incorporating vitamin D supplementation trials, longitudinal monitoring, and exercise-induced perfusion protocols may help determine whether microvascular perfusion improves directly in response to corrected vitamin D status.

This study has several limitations. First, the cross-sectional design does not allow causal interpretation of the relationship between serum 25-hydroxyvitamin D concentration and skeletal muscle microvascularity. Second, although the sample size was adequate for the primary analyses, a multicenter or longitudinal design could provide more robust evidence by evaluating changes in vascular index over time. Additionally, classification of participants was based on a single serum 25-hydroxyvitamin D measurement, which may not fully capture long-term vitamin D status and could contribute to residual misclassification. The study population consisted of relatively young and healthy adults, which may have reduced inter-individual heterogeneity. Finally, UMA-derived vascular index provides an indirect assessment of microvascular perfusion; although measurement conditions were highly standardized, ultrasound-based parameters inherently carry some degree of technical variability. In addition, although imaging parameters were standardized to ensure consistency, the use of fixed sensitivity settings across different muscle groups and body compositions may have introduced a degree of systematic measurement bias, which should be considered when interpreting the absolute magnitude of vascular index values. Importantly, key determinants of vitamin D status and muscle perfusion, including habitual sun exposure and physical activity, were assessed solely by self-report and not quantified using standardized or validated questionnaires, which may have resulted in residual confounding. Because sun exposure and physical activity were assessed only by self-report and not quantified using standardized instruments, these variables were not included as covariates in the multivariable regression models. Although multivariable regression analyses indicated that age, sex, body mass index, and muscle cross-sectional area did not materially alter these associations, residual systemic confounding cannot be fully excluded and should be considered when interpreting the strength of the observed correlations. Future studies incorporating functional assessments and longitudinal follow-up may offer deeper insight into the clinical relevance of these findings. Nevertheless, the consistency of vascular index measurements across three anatomically distinct muscle groups and the strong correlations observed support the robustness of the detected associations.

## 5. Conclusions

In conclusion, serum 25-hydroxyvitamin D concentration was associated with skeletal muscle microvascularity, as assessed by quantitative ultrasound imaging. The observed relationship between vitamin D status and microvascular perfusion suggests that adequate vitamin D levels may contribute to improved muscle perfusion and metabolic support. UMA provides a dynamic, non-invasive approach for evaluating microvascular alterations related to vitamin D status and may serve as a valuable tool in future musculoskeletal research. Given its accessibility, cost-effectiveness, and sensitivity to early microcirculatory changes, UMA may represent a complementary imaging modality for assessing muscle health in both research and clinical settings. Prospective studies incorporating functional outcomes and vitamin D supplementation protocols are warranted to further clarify the temporal and causal relationships underlying these observations.

## Figures and Tables

**Table 1:** Demographic and muscle characteristics of participants (*n* = 141) (values expressed as median (min–max) and mean ± SD).

		25(OH)D Concentrations < 20 ng/mL	25(OH)D Concentrations > 20 ng/mL	*p*-Value
Gender (female), *n* (%) *		44/71 (62.0)	46/70 (65.7)	0.644
Age, (years) **		24 (19–47)	33 (20–46)	<0.001
Muscle CSA (Female) **	**FCR**	400 mm^2^ (359–422) 402 ± 17	399 mm^2^ (364–452) 406 ± 22	0.904
	**BB (short head)**	422 mm^2^ (401–445) 430 ± 18	424 mm^2^ (407–482) 438 ± 24	0.762
	**TA**	400 mm^2^ (361–422) 409 ± 20	400 mm^2^ (365–432) 412 ± 21	0.728
Muscle CSA (Male) **	**FCR**	444 mm^2^ (416–478) 448 ± 22	440 mm^2^ (424–480) 452 ± 26	0.637
	**BB (short head)**	476 mm^2^ (455–508) 482 ± 21	475 mm^2^ (456–518) 489 ± 27	0.331
	**TA**	452 mm^2^ (423–484) 462 ± 24	443 mm^2^ (425–484) 458 ± 28	0.597

* Chi-Square Tests, data are presented as counts, with percentages in brackets; ** Mann–Whitney U test, data are presented as median (minimum-maximum). 25(OH)D: 25-hydroxyvitamin D. CSA: Cross Sectional Area BB: biceps brachii, FCR: Flexor carpi radialis, TA: Tibialis anterior. CSA values are presented in mm^2^ and represent the anatomical cross-sectional area of each muscle.

**Table 2:** Comparison of UMA parameters of FCR, BB, TA muscles according to the 25(OH)D concentrations (*n* = 141).

	25(OH)D Concentrations < 20 ng/mL	25(OH)D Concentrations > 20 ng/mL	*p*-Value
**FCR**	0.90 (0.50–1.20)	1.50 (1.10–2.40)	<0.001
**BB (short head)**	1.00 (0.60–1.30)	1.70 (1.20–2.70)	<0.001
**TA**	0.90 (0.50–1.20)	1.40 (1.10–2.20)	<0.001

Mann–Whitney U test, data are presented as median (minimum-maximum). 25(OH)D: 25-hydroxyvitamin D. BB: biceps brachii, FCR: flexor carpi radialis, TA: tibialis anterior.

**Table 3:** ROC analysis results of UMA parameters for muscles.

	AUC (95% CI)	*p*-Value	Cut-Off	Sensitivity	Specificity
**FCR**	0.983 (0.968–0.997)	**<0.001**	≥1.15	97.1	87.3
**BB (short head)**	0.992 (0.984–1)	**<0.001**	≥1.25	95.7	97.2
**TA**	0.988 (0.976–1)	**<0.001**	≥1.15	94.3	94.4

AUC: Area under the curve, 95% CI: 95% confidence interval.

## Data Availability

The datasets generated and analyzed during the current study are available from the corresponding author upon reasonable request. Access to these data is restricted to protect participant confidentiality and comply with ethical regulations.

## Abbreviations

AUC	Area Under the Curve
BB	biceps brachii
CT	Computed Tomography
DEXA	Dual-Energy X-Ray Absorptiometry
FCR	Flexor Carpi Radialis
MRI	Magnetic Resonance Imaging
NO	Nitric Oxide
ROC	Receiver Operating Characteristic
SMI	Superb Microvascular Imaging
UMA	Ultra Micro Angiography
US	Ultrasound
UV	Ultraviolet
TA	tibialis anterior
VI	Vascular Index
